# Supplementary material for: From sequence to enzyme mechanism using multi-label machine learning
Source: BMC Bioinformatics. 2014 May 19;15:150. doi: 10.1186/1471-2105-15-150 (PMC4229970; doi:10.1186/1471-2105-15-150)
Supplement: Additional file 2 — Java code of ml2db. Additional file ml2db_code.tar.gz contains the Java source code to run the multi-label machine learning experiments and save the results to database. The code’s Javadoc is included. [file 1471-2105-15-150-S2.zip › additional file 2/ml2db/ml2db/doc/main/ml2db/mulan/mulanarff/Statistics.html]

Statistics


JavaScript is disabled on your browser.


- Overview
- Package
- Class
- Use
- Tree
- Deprecated
- Index
- Help

- Prev Class
- Next Class

- Frames
- No Frames

- All Classes

- Summary:
- Nested |
- Field |
- Constr |
- Method

- Detail:
- Field |
- Constr |
- Method


main.ml2db.mulan.mulanarff

## Class Statistics

- java.lang.Object
- - main.ml2db.mulan.mulanarff.Statistics

- All Implemented Interfaces:
  :   java.io.Serializable

  ---

    

  ```
  public class Statistics
  extends java.lang.Object
  implements java.io.Serializable
  ```

  Class exteded from Mulan
   Class for calculating statistics of a multilabel
  dataset


   BibTeX:

  ```
   @article{tsoumakas+katakis:2007,
      author = {G. Tsoumakas, I. Katakis},
      journal = {International Journal of Data Warehousing and Mining},
      pages = {1-13},
      title = {Multi-Label Classification: An Overview},
      volume = {3},
      number = {3},
      year = {2007}
   }
  ```

   Valid options are:

  ```
   -F <filename>
    The filename (including full path) of the multilabel mlData set).
  ```

  ```
   -L <number of labels>
    Number of labels.
  ```

  Version:
  :   $Revision: 1.4 $, 12 Apr 2012

  Author:
  :   Grigorios Tsoumakas, Robert Friberg, Luna De Ferrari ldf(at)st-andrews.ac.uk or
      luna.deferrari@st-andrews.ac.uk

  See Also:
  :   Serialized Form

- - ### Constructor Summary

    Constructors

    Constructor and Description

    `Statistics()`
  - ### Method Summary

    Methods

    | Modifier and Type | Method and Description |
    | --- | --- |
    | `double[][]` | `calculateCoocurrence(mulan.data.MultiLabelInstances mdata)` This method calculates and prints a matrix with the coocurrences of   pairs of labels |
    | `double[][]` | `calculatePhi(mulan.data.MultiLabelInstances dataSet)` Calculates phi correlation |
    | `void` | `calculateStats(mulan.data.MultiLabelInstances mlData)` calculates various multilabel statistics, such as label cardinality,   label density and the set of distinct labels along with their frequency |
    | `double` | `cardinality()` returns the label cardinality of the dataset |
    | `double` | `density()` returns the label density of the dataset |
    | `double[]` | `getCardinalityDistribution()` |
    | `double[]` | `getExamplesPerLabel()` |
    | `double` | `getLabelCardinality()` |
    | `double` | `getLabelDensity()` |
    | `java.util.HashMap<mulan.data.LabelSet,java.lang.Integer>` | `getLabelsets()` |
    | `int` | `getNumAttributesWithExamples()` |
    | `int` | `getNumClassLabelsWithExamples()` |
    | `int` | `getNumInstances()` |
    | `int` | `getNumLabels()` |
    | `int` | `getNumNominal()` |
    | `int` | `getNumNumeric()` |
    | `int` | `getNumPredictors()` |
    | `double[][]` | `getPhi()` |
    | `double[]` | `getPhiHistogram()` Calculates a histogram of phi correlations |
    | `java.util.HashMap<mulan.data.LabelSet,java.lang.Integer>` | `labelCombCount()` returns the HashMap containing the distinct labelsets and their frequencies |
    | `int` | `labelFrequency(mulan.data.LabelSet x)` returns the frequency of a labelset in the dataset |
    | `java.util.Set<mulan.data.LabelSet>` | `labelSets()` returns a set with the distinct labelsets of the dataset |
    | `void` | `printPhiCorrelations()` Prints out phi correlations |
    | `void` | `printPhiDiagram(double step)` This method prints data, useful for the visualization of Phi per dataset. |
    | `double[]` | `priors()` returns the prior probabilities of the labels |
    | `void` | `setNumAttributesWithExamples(mulan.data.MultiLabelInstances mlData)` |
    | `void` | `setNumLabelsWithExamples(mulan.data.MultiLabelInstances mlData)` |
    | `int[]` | `topPhiCorrelatedLabels(int labelIndex, int k)` Returns the indices of the labels that have the strongest phi correlation with the label which is given as a parameter. |
    | `java.lang.String` | `toString()` returns various multilabel statistics in textual representation |
    | `int[]` | `uncorrelatedLabels(int labelIndex, double bound)` returns the indices of the labels whose phi coefficient values lie between -bound <= phi <= bound |

    - ### Methods inherited from class java.lang.Object

      `equals, getClass, hashCode, notify, notifyAll, wait, wait, wait`

- - ### Constructor Detail


    - #### Statistics

      ```
      public Statistics()
      ```
  - ### Method Detail


    - #### calculateCoocurrence

      ```
      public double[][] calculateCoocurrence(mulan.data.MultiLabelInstances mdata)
      ```

      This method calculates and prints a matrix with the coocurrences of   
      pairs of labels

      Parameters:
      :   `mdata` - a multi-label data set

      Returns:
      :   a matrix of co-occurences


    - #### calculatePhi

      ```
      public double[][] calculatePhi(mulan.data.MultiLabelInstances dataSet)
                              throws java.lang.Exception
      ```

      Calculates phi correlation

      Parameters:
      :   `dataSet` - a multi-label dataset

      Returns:
      :   a matrix containing phi correlations

      Throws:
      :   `java.lang.Exception`


    - #### calculateStats

      ```
      public void calculateStats(mulan.data.MultiLabelInstances mlData)
      ```

      calculates various multilabel statistics, such as label cardinality,   
      label density and the set of distinct labels along with their frequency

      Parameters:
      :   `mlData` - a multi-label dataset


    - #### cardinality

      ```
      public double cardinality()
      ```

      returns the label cardinality of the dataset

      Returns:
      :   label cardinality


    - #### density

      ```
      public double density()
      ```

      returns the label density of the dataset

      Returns:
      :   label density


    - #### getCardinalityDistribution

      ```
      public double[] getCardinalityDistribution()
      ```

      Returns:
      :   m\_cardinalityDistribution


    - #### getExamplesPerLabel

      ```
      public double[] getExamplesPerLabel()
      ```

      Returns:
      :   m\_examplesPerLabel


    - #### getLabelCardinality

      ```
      public double getLabelCardinality()
      ```

      Returns:
      :   m\_labelCardinality


    - #### getLabelDensity

      ```
      public double getLabelDensity()
      ```

      Returns:
      :   m\_labelDensity


    - #### getLabelsets

      ```
      public java.util.HashMap<mulan.data.LabelSet,java.lang.Integer> getLabelsets()
      ```

      Returns:
      :   m\_labelsets


    - #### getNumAttributesWithExamples

      ```
      public int getNumAttributesWithExamples()
      ```

      Returns:
      :   m\_numAttributesWithExamples


    - #### getNumClassLabelsWithExamples

      ```
      public int getNumClassLabelsWithExamples()
      ```

      Returns:
      :   m\_numLabelsWithExamples


    - #### getNumInstances

      ```
      public int getNumInstances()
      ```

      Returns:
      :   m\_numInstances


    - #### getNumLabels

      ```
      public int getNumLabels()
      ```

      Returns:
      :   m\_numLabels


    - #### getNumNominal

      ```
      public int getNumNominal()
      ```

      Returns:
      :   m\_numNominal


    - #### getNumNumeric

      ```
      public int getNumNumeric()
      ```

      Returns:
      :   m\_numNumeric


    - #### getNumPredictors

      ```
      public int getNumPredictors()
      ```

      Returns:
      :   m\_numPredictors


    - #### getPhi

      ```
      public double[][] getPhi()
      ```

      Returns:
      :   m\_phi


    - #### getPhiHistogram

      ```
      public double[] getPhiHistogram()
      ```

      Calculates a histogram of phi correlations

      Returns:
      :   an array with phi correlations


    - #### labelCombCount

      ```
      public java.util.HashMap<mulan.data.LabelSet,java.lang.Integer> labelCombCount()
      ```

      returns the HashMap containing the distinct labelsets and their
      frequencies

      Returns:
      :   HashMap with distinct labelsest and their frequencies


    - #### labelFrequency

      ```
      public int labelFrequency(mulan.data.LabelSet x)
      ```

      returns the frequency of a labelset in the dataset

      Parameters:
      :   `x` - a labelset

      Returns:
      :   the frequency of the given labelset


    - #### labelSets

      ```
      public java.util.Set<mulan.data.LabelSet> labelSets()
      ```

      returns a set with the distinct labelsets of the dataset

      Returns:
      :   set of distinct labelsets


    - #### printPhiCorrelations

      ```
      public void printPhiCorrelations()
      ```

      Prints out phi correlations


    - #### printPhiDiagram

      ```
      public void printPhiDiagram(double step)
      ```

      This method prints data, useful for the visualization of Phi per dataset.
      It prints int(1/step) + 1 pairs of values. The first value of each pair
      is the phi value and the second is the average number of labels that
      correlate to the rest of the labels with correlation higher than the
      specified phi value;

      Parameters:
      :   `step` - the phi value increment step


    - #### priors

      ```
      public double[] priors()
      ```

      returns the prior probabilities of the labels

      Returns:
      :   array of prior probabilities of labels


    - #### setNumAttributesWithExamples

      ```
      public void setNumAttributesWithExamples(mulan.data.MultiLabelInstances mlData)
      ```

      Parameters:
      :   `data` - to calculate the statistics for


    - #### setNumLabelsWithExamples

      ```
      public void setNumLabelsWithExamples(mulan.data.MultiLabelInstances mlData)
      ```

      Parameters:
      :   `data` - to calculate the statistics for


    - #### topPhiCorrelatedLabels

      ```
      public int[] topPhiCorrelatedLabels(int labelIndex,
                                 int k)
      ```

      Returns the indices of the labels that have the strongest phi correlation
      with the label which is given as a parameter. The second parameter is the
      number of labels that will be returned.

      Parameters:
      :   `labelIndex` -
      :   `k` -

      Returns:
      :   the indices of the k most correlated labels


    - #### toString

      ```
      public java.lang.String toString()
      ```

      returns various multilabel statistics in textual representation

      **Overrides:**
      :   `toString` in class `java.lang.Object`


    - #### uncorrelatedLabels

      ```
      public int[] uncorrelatedLabels(int labelIndex,
                             double bound)
      ```

      returns the indices of the labels whose phi coefficient values lie
      between -bound <= phi <= bound

      Parameters:
      :   `labelIndex` -
      :   `bound` -

      Returns:
      :   the indices of the labels whose phi coefficient values lie
          between -bound <= phi <= bound


- Overview
- Package
- Class
- Use
- Tree
- Deprecated
- Index
- Help

- Prev Class
- Next Class

- Frames
- No Frames

- All Classes

- Summary:
- Nested |
- Field |
- Constr |
- Method

- Detail:
- Field |
- Constr |
- Method
